# Sustainability, productivity, profitability and soil health with conservation agriculture based sustainable intensification of oilseed brassica production system

**DOI:** 10.1038/s41598-021-92801-z

**Published:** 2021-06-28

**Authors:** R. S. Jat, R. L. Choudhary, H. V. Singh, M. K. Meena, V. V. Singh, P. K. Rai

**Affiliations:** ICAR-Directorate of Rapeseed-Mustard Research, Bharatpur, Rajasthan India

**Keywords:** Climate sciences, Environmental sciences

## Abstract

Conservation agriculture (CA) practices are getting space world-wide to answer many emerging challenges like; declining factor productivity, deteriorating soil health, water scarcity, climate change, and farm profitability and sustainability. Oilseed brassica (Indian mustard, *Brassica juncea* L.), a winter oilseed grown under rainfed agro-ecosystem is vulnerable to low yields, high production cost, degrading soil and water quality, and climatic vagaries. The present study was undertaken on CA-based sustainable intensification of Indian mustard for enhancing inputs efficiencies, farm profitability and sustainability. Permanent beds with residue retention (PB + R) improved mustard equivalent yield (11.4%) and system grain yield (10.6%) compared with conventional tillage without residue (CT − R). Maize–mustard rotation (Mz–M) increased system grain yield (142.9%) as well as mustard equivalent yield (60.7%) compared with fallow-mustard (F-M). Mz–M system under PB + R increased sustainable yield index (376.5%), production efficiency (177.2%), economic efficiency (94%) and irrigation water productivity (66%) compared with F-M under CT − R. PB + R increased soil organic carbon (SOC) stock at 0–15 cm (17.7%) and 15–30 cm (29.5%) soil depth compared with CT − R. Addition of green gram in rotation with mustard improved SOC at 0–15 cm (27.4%) and 15–30 cm (20.5%) compared with F-M system. CA-based cluster bean-mustard/GG-M system increased N productivity, whereas, P and K productivity improved with Mz–M system compared with F-M under CT − R. Thus, CA-based Mz–M system should be out-scaled in the traditional rainfed fallow-mustard system to improve the farm production and income on holistic basis to make the country self-sufficient in edible oils.

## Introduction

Conservation agriculture is being practiced over 125 million hectares world-wide^1^ and several reports of reduced production costs, improved water-use efficiency, and sustained or increased crop productivity across the globe in the present era of resource degradation and climate change have been attributed to the practice^2–8^. Sustainable intensification of crops and cropping systems, as one of the principles of conservation agriculture, hold a lot of potential to withstand climatic anomaly, price fluctuation, balanced food supply, natural resource degradation, and fertilizer and pesticide dependence. Conservation agriculture-based system intensification in the vulnerable semi-arid tropics provides opportunities to conserve and utilize the fatiguing natural resources more efficiently, increase resilience to anomalous climatic events, and to increase productivity and farmers’ profitability while minimizing production cost and energy use. Besides this, crop intensification improves the nutritional security of the farm households and reduces the risk of total crop failure in unfavorable or erratic weather situations^9^. In rice–wheat system, CA-based sustainable intensification increased productivity (10–17%) and profitability (24–50%) at less irrigation water (15–71%), energy (17–47%) and carbon footprints than conventional practices^10^. The benefits of CA based crop management practices appraised across the globe^11,12^, even though, the scope of adoption in rainfed smallholder farming systems remained contentious due to ecological and socio-economic constraints^12^. Considering various arguments, CA must obviously be adapted to local agro-ecological conditions, and farmer capabilities and preferences. Fundamentally, to derive maximum benefit from CA, location-specific appropriate crop rotations and system-based CA practices need to be standardized^13,14^.

India is the 5th largest vegetable oil economy in the world, accounting for 7.4% oilseeds, 5.8% oils and 6.1% oil meal production, and 9.3% consumption of edible oils^15^. Vegetable oils account for the second most important agricultural economy in India next to cereals, growing at a pace of 4.1% per annum in the last three decades. Despite being the third largest producer (11.3%) of rapeseed and mustard in the world, after Canada and China, India meets 60% of the domestic edible oil requirements through imports and is ranked the 7th largest importer. The country needs 25 MT of edible oils to meet its requirement at the current consumption level of 19 kg per person per annum. Indian mustard holds sizable contribution, however, the productivity levels are 2/3rd of the world level due to large scale cultivation under rainfed situation where crop often encounter biotic and abiotic stresses, and resources crunch^15,16^. The conventional rapeseed and mustard production system in India largely suffers due to excessive tillage, poor crop establishment and monotonous cropping system which exaggerate the resource degradation and cost of production^17,18^. Indian mustard dominantly grown as winter oilseed under fragile rainfed ecology with intensive land preparation involving multiple passes of discs/tine harrows and planking to create a friable seedbed. Undesired excessive tillage practices for field preparation^19^ leads to breakdown of soil organic carbon^20,21^ which decline the soil fertility and microbial population. It also leads to early exhaustion of soil moisture which is a major apprehension in the rainfed ecology. CA-based crop management practices are mostly being scaled-out in major cereal based cropping system like, rice/maize-wheat systems in India, and very less efforts being made in oilseed/pulse systems. There is need to develop an alternative holistic management strategy based on ecotypic and conservation agriculture principles for enhanced system capacity, biomass production, and energy-use efficiency and reduction in carbon footprints.

Indian mustard, a dominant and versatile oilseed crop of the semi-arid tropics, needs incessant system-based approaches at appropriate scale to exploit the metabolic potential of cultivars while enduring the growing climatic stresses. CA-based sustainable intensification of the traditional fallow-mustard system in the rainfed ecology holds promises to address the shortfall of oilseed and edible oil in the country and reduce the import burden. The present study will provide insights of (1) CA-based system intensification of Indian mustard production under rainfed ecologies, (2) enhanced inputs and output efficiencies, and (3) sustainability, economic viability and soil health in CA-based Indian mustard systems.

## Results and discussion

### Production and economics

Conservation tillage practice, PB + R being on par with ZT + R recorded markedly higher (p = 0.05) mustard seed yield compared with CT − R (Table 1). Permanent bed with residue produced maximum mustard seed yield (3.0 Mg ha^−1^) followed by zero tillage with residue (2.8 Mg ha^−1^) and conventional tillage without residue (2.6 Mg ha^−1^). PB + R, though, on par with ZT + R increased mustard seed yield by 15.4% (3-year mean) over the CT − R. Intensification of mustard-based cropping systems through cluster bean (CB-M), green gram (GG-M) and maize (Mz–M) crops during the rainy season (July–September) increased mustard seed yield (3-year mean) compared with fallow-mustard (farmers practice in this region). On the other hand, addition of pearl millet (PM-M) and sesame (S-M) during the rainy season lowered mustard seed yield. The highest mustard seed yield was recorded in maize-mustard cropping system (3.1 Mg ha^−1^) (Table 1) followed by CB-M and GG-M. The seed yield of mustard and other crops in the system improved in the permanent beds might be due to better soil physicochemical and biological properties, and nutrient availability that are related to tillage and residue management practices. Higher productivity and profitability in CA-based management was reported in mustard^22^ and sesame-based cropping system^23^ compared with mono-cropping. Permanent bed planting ensured higher mustard yield due to complementary border effects^24^ which are more under residue retention than conventional tillage without residue. Advantage of CA in rice–wheat and maize–wheat systems was reported to enhance the crop productivity, water productivity, profitability, and water saving, compared with CT-based systems^25^.

**Table 1:** Crop yields, system productivity and economics of CA-based Indian mustard systems (3 years mean).

Treatments^A^		Mustard seed yield (Mg ha^−1^)	Rainy crops yield (Mg ha^−1^)	System grain yield (Mg ha^−1^)	Mustard equivalent yield (Mg ha^−1^)	Net returns (US$ ha^−1^)^B^	REE (%)^C^
**Tillage practices**							
PB + R		3.0^a†^	1.4^a^	4.3^a^	3.9^a^	1720^a^	20.3
ZT + R		2.8^ab^	1.2^b^	4.0^b^	3.5^b^	1518^b^	6.2
CT − R		2.6^b^	1.2^b^	3.9^b^	3.5^b^	1430^b^	–
**Cropping systems**							
F-M		2.8^c^	0	2.8^e^	2.8^e^	1291^d^	–
CB-M		2.9^b^	1.0^c^	3.9^b^	3.9^c^	1777^c^	37.7
GG-M		2.9^b^	0.8^d^	3.8^c^	4.2^b^	1854^b^	43.7
Mz–M		3.1^a^	3.7^a^	6.8^a^	4.5^a^	1982^a^	53.6
PM-M		2.6^d^	1.5^b^	4.1^b^	3.2^d^	1247^de^	– 3.4
S-M		2.4^e^	0.6^e^	3.0^d^	3.2^d^	1185^e^	– 8.2

^†^Means followed by a similar lowercase letters within a column are not significantly different at 0.05 level of probability using DMRT.

^A^Refer to Table 7 for treatment description.

^B^US$ = 70.34 INR.

^C^Relative economic efficiency.

**Figure 1:**
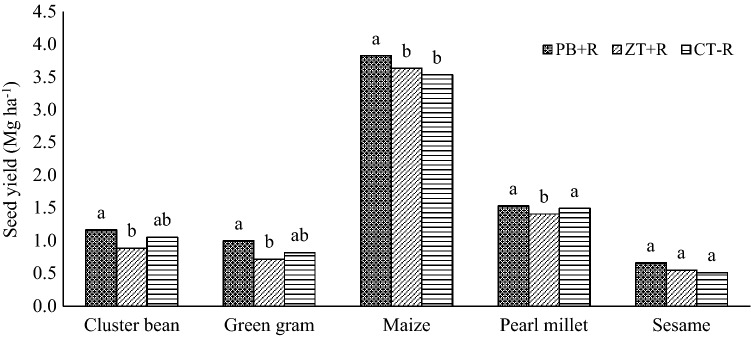
Yield of rainy season crops in Indian mustard-based cropping systems under different CA practices (mean of 3 years).

Rainy season crops recorded higher seed yield (3-year mean across the crops) under PB + R (1.4 Mg ha^−1^) compared with ZT + R and CT − R (Table 1). Among the cropping systems, maize was found most productive (3.7 Mg ha^−1^) followed by pearl millet, cluster bean, green gram and sesame with The average seed yield of cluster bean, green gram, maize and pearl millet improved markedly (p = 0.05) in the PB + R compared with ZT + R, though, found on par with CT − R (Fig. 1). Sesame did not show any significant difference in seed yield due to tillage and residue management practices.

Conservation tillage practice, PB + R increased overall system grain yield (yield of rainy crops and mustard) and recorded higher (4.3 Mg ha^−1^) (3-year mean) (p = 0.05) (Table 2) compared with ZT + R (4.0 Mg ha^−1^) and CT − R (3.9 Mg ha^−1^) (Table 1). ZT + R did not show significant variation in system grain yield over the CT − R. PB + R increased system grain yield by 10.3 and 7.5% over CT − R and ZT + R, respectively. The highest system grain yield was recorded in Mz–M system (6.8 Mg ha^−1^) followed by PM-M, CB-M, GG-M, S-M and the lowest in the F-M cropping system (2.8 Mg ha^−1^). Mz–M system increased system grain yield by 142.9% over the F-M system which is the most popular system among the regional farmers. The system productivity in terms of mustard equivalent yield (seed yield of rainy season crops converted to mustard seed yield) was markedly higher (3-year mean) under the PB + R (3.9 Mg ha^−1^) compared with ZT + R and CT − R (Table 1). Mustard equivalent yield increased by 11.4% under PB + R over the ZT + R and CT − R. The system productivity in terms of mustard equivalent yield of different cropping systems revealed the highest of Mz–M (4.5 Mg ha^−1^) followed by GG-M, CB-M, PM-M and S-M system and the lowest in the fallow-mustard system (2.8 Mg ha^−1^). Mz–M system increased system productivity (mustard equivalent yield) by 60.7% over fallow-mustard system. The system grain yield and mustard equivalent yield increased in the permanent beds might be due to favorable soil–plant–environment continuum in the permanent beds complementing with crop residues. CA-based system productivity enhancement were also reported to increase in mustard under rice-mustard system^26,27^.

**Table 2:** ANOVA for ^a^mustard seed yield (Mg ha^−1^), ^b^rainy crops yield (Mg ha^−1^), ^c^system grain yield (Mg ha^−1^), ^d^mustard equivalent yield (Mg ha^−1^), and ^e^net returns (US$ ha^−1^).

Source	LSD (p = 0.05)					R^2^				
	MSY^a^	RCY^b^	SGY^c^	MEY^d^	NR^e^	MSY	RCY	SGY	MEY	NR
Tillage practices	0.2	0.03	0.1	0.1	136	0.92	0.99	0.99	0.98	0.98
Cropping systems	0.1	0.1	0.2	0.2	73					

The conservation tillage practice, PB + R showed markedly higher (3-year mean) (p = 0.05) (Table 2) net profit (1720 US$ ha^−1^) compared with ZT + R (1518 US$ ha^−1^) and CT − R (1430 US$ ha^−1^) (Table 1). PB + R increased net returns by 20.3 and 13.3% over CT − R and ZT + R, respectively. Crop intensification in the rainy season increased the overall net profit over the fallow-mustard system (farmers practice). The highest net return was recorded in Mz–M system (1982 US$ ha^−1^) followed by GG-M, CB-M, PM-M, S-M and the lowest profit was accrued in the fallow-mustard system (1291 USD ha^−1^). Mz–M system increased net return by 53.5% over the fallow-mustard system. PB + R and ZT + R showed the higher REE (20.3 and 6.2%, respectively) in comparison to CT − R (Farmers practice) (Table 1). Among the cropping systems, REE increased with the Mz–M (53.6%), GG-M (43.7%) and CB-M (37.7%) systems, However, decreased REE in the PM-M (− 3.4%) and S-M (− 8.2%) mainly due to low mustard seed yield in these systems compared with fallow-mustard system.

**Figure 2:**
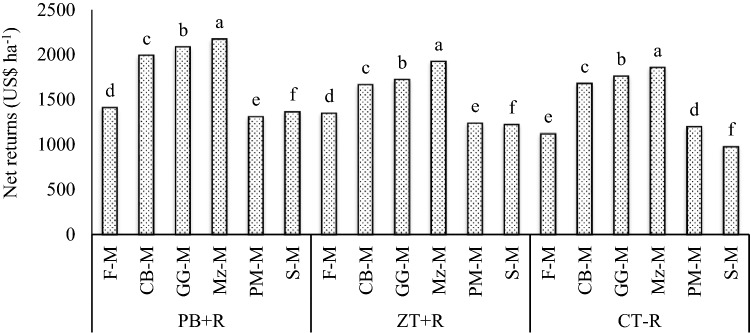
Net return of different CA-based Indian mustard systems (3-year mean).

The interaction effects between CA practices and cropping systems (Fig. 2) showed highest net returns (2169 US$ ha^−1^) in Mz–M system under PB + R followed by GG-M (2083 US$ ha^−1^) and CB-M (1990 US$ ha^−1^) systems. Mz–M system under PB + R increased net return by 94.2% over F-M system under CT − R (farmers practice) (3-year mean) (Fig. 2). Increase in net returns of Mz–M system in PB + R might be due to better yield of mustard in the system and that to mainly in PB + R due to added advantages of tillage practices and residue retention. Combined, these results clearly demonstrate the potential of CA towards sustainable intensification of crop production to improve future household income and food security^28,29^.

### Sustainability and input use efficiencies

**Figure 3:**
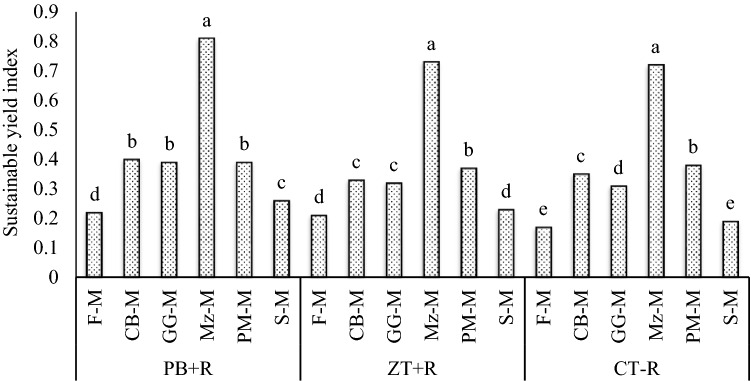
Sustainable yield index of different CA-based Indian mustard-based systems (3-year mean).

The conservation agriculture practice, PB + R was found more sustainable compared with ZT + R and CT − R (3-year mean). PB + R recorded higher (p = 0.05) (Table 4) sustainable yield index (0.41) exceeding ZT + R and CT − R by 13.9% (Table 3). Among the mustard-based cropping systems intensification, Mz–M system recorded the highest SYI (0.75), whereas, the least was found in the fallow-mustard system (0.20) (Table 3). The interaction effects between tillage practices and cropping systems (Fig. 3) showed that Mz–M system recorded significantly higher SYI under PB + R (0.81) compared with other systems. Mz–M system under PB + R increased SYI by 376.5% compared with F-M under CT − R (farmers practice). Permanent beds with residue under maize-mustard cropping system reported highest SYI due to higher yields, improved soil conditions, organic carbon build up, and residue incorporation^27,30,31^. It might also be due to higher assimilation of metabolizable C and N in crop plants due to residue retention, increased root biomass, and root absorption^32^.

**Table 3:** Sustainability, production and economic efficiency, and water productivity under different CA-based Indian mustard systems (mean of 3 years).

Treatments^A^	SYI^B^	PE (kg grain day^−1^)^C^	EE (US$ day^−1^)^D^	IWP (kg grain M^−3^)^E^
**Tillage practices**				
PB + R	0.41^a†^	15.7^a^	6.4^a^	3.14^a^
ZT + R	0.36^b^	14.4^b^	5.6^b^	2.32^b^
CT − R	0.36^b^	14.1^b^	5.3^b^	2.25^b^
**Cropping systems**				
F-M	0.20^f^	10.2^e^	4.8^d^	2.49^c^
CB-M	0.36^c^	14.3^b^	6.6^c^	2.86^a^
GG-M	0.34^d^	13.7^c^	6.9^b^	2.75^b^
Mz–M	0.75^a^	24.6^a^	7.3^a^	2.94^a^
PM-M	0.38^b^	14.8^b^	4.6^de^	2.2^d^
S-M	0.23^e^	10.9^d^	4.4^e^	2.17^d^

^†^Means followed by a similar lowercase letters within a column are not significantly different at 0.05 level of probability using DMRT.

^A^Refer Table 7 for treatment description.

^B^Sustainable yield index.

^C^Production efficiency.

^D^Economic efficiency.

^E^Irrigation water productivity.

**Figure 4:**
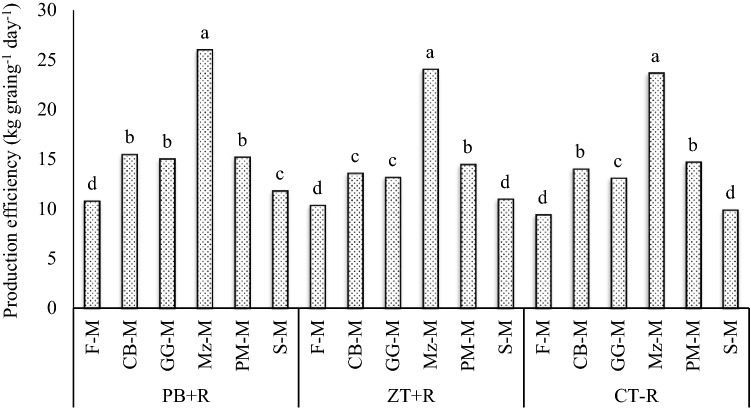
Production efficiency of CA-based Indian mustard systems (3-year mean).

Production per day spread over the crop duration (3-year mean) increased (p = 0.05) (Table 4) evidently under the PB + R compared with ZT + R and CT − R (Table 3). Production efficiency was highest under PB + R (15.7 kg grain day^−1^) which was 9.0 and 11.3% more compared with ZT + R and CT − R, respectively. Addition of rainy season crops in the system increased production efficiency significantly compared with fallow-mustard (farmer’s practices). Mz–M system recorded the highest PE (24.6 kg grain day^−1^) followed by PM-M (14.8 kg grain day^−1^), CB-M (14.3 kg grain day^−1^), GG-M (13.7 kg grain day^−1^) and S-M (10.9 kg grain day^−1^), and the lowest was recorded in the F-M (10.2 kg grain day^−1^). Mz–M system increased production efficiency by 141.2% over the fallow-mustard system. Interaction effects showed that the Mz–M system also recorded significantly higher production efficiency under PB + R (26 kg grain day^−1^) compared with other systems and tillage practices (3-year mean) (Fig. 4). Mz–M system under PB + R increased PE by 177.2% over the F-M under CT − R (farmers practice).

**Table 4:** ANOVA for ^a^sustainable yield index.

Source	LSD (p = 0.05)				R^2^			
	SYI^a^	PE^b^	EE^c^	IWP^d^	SYI	PE	EE	IWP
Tillage practices	0.03	0.77	0.50	0.14	0.99	0.99	0.97	0.98
Cropping systems	0.02	0.47	0.27	0.08				

^b^Production efficiency.

^c^Economic efficiency.

^d^Irrigation water productivity.

**Figure 5:**
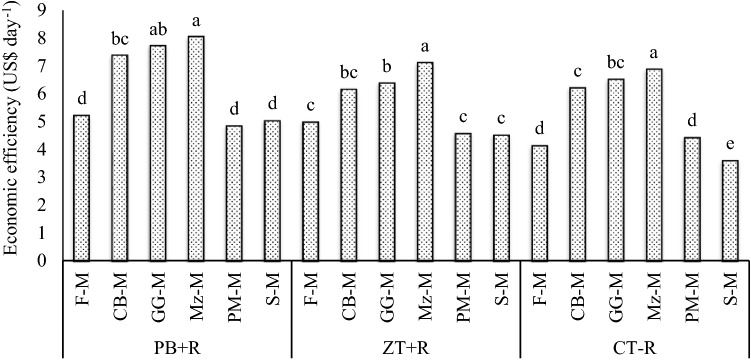
Economic efficiency of CA-based Indian mustard systems (3-year mean).

Economic efficiency in terms of US$ day^−1^ (3-year mean) was highest (p = 0.05) (Table 4) in the PB + R (6.4 US$ day^−1^) compared with ZT + R (5.6 US$ day^−1^) and CT − R (5.3 US$ day^−1^). The EE increased by 14.3, 20.8% over the ZT + R and CT − R, respectively (Table 3). Among different cropping systems, highest economic efficiency was achieved in Mz–M system (7.3 US$ day^−1^) followed by GG-M (6.9 US$ day^−1^) and CB-M (6.6 US$ day^−1^). The EE decreased in case of PM-M (4.6 US$ day^−1^) and S-M (4.4 US$ day^−1^) compared with the F-M system (4.8 US$ day^−1^). The Mz–M system increased the economic efficiency by 52.1% compared with fallow-mustard system (farmers practice). Again, the interaction effects showed that Mz–M system recorded higher EE under PB + R (8 US$ day^−1^) compared with other systems and tillage practices (3-year mean) (Fig. 5). Mz–M system under PB + R increased EE by 94% over the F-M under CT − R (farmers practice). Production efficiency of Mz–M system increased might be due to higher system grain yield compared with other systems in the same crop duration of 270 days per year. Economic efficiency of Mz–M and GG-M systems were higher due to higher grain yield and fetched remunerative sale price besides the reduced costs of production in comparison to farmer’s practice. The EE declines in the PM-M and S-M, systems, compared with F-M might be due to negative rotation effects and the lower seed yields of mustard under these systems. Similar results were also reported by^19,29^.

**Figure 6:**
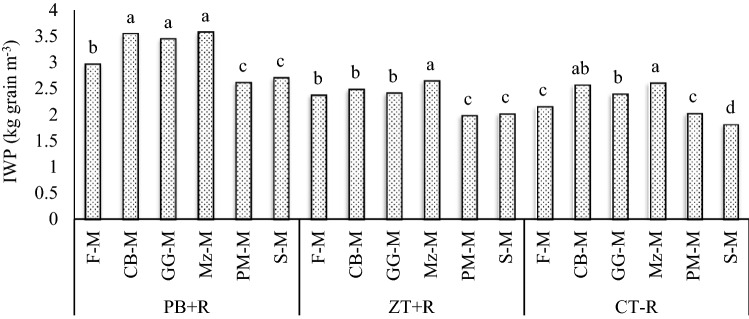
Irrigation water productivity of CA-based Indian mustard systems (3-year mean).

The conservation tillage practice, PB + R recorded the highest (p = 0.05) (Table 4) irrigation water productivity in terms of kg grain per M^3^ of water (3-year mean) compared with ZT + R and CT − R (Table 3). PB + R recorded IWP of 3.14 kg grain M^−3^ which was 35.4 and 39.6% over the ZT + R (2.32 kg grain M^−3^) and CT − R (2.25 kg grain M^−3^) values, respectively. Addition of one more crop in the rainy season increased the system IWP markedly (3-year mean) and recorded the highest value in the Mz–M system (2.94 kg grain M^−3^). The Mz–M system increased IWP by 18.1% compared with the fallow-mustard system (farmers practice). The interaction effects between tillage practices and cropping systems (3-year mean) (Fig. 6) showed that the Mz–M, system under PB + R recorded the highest IWP (3.57 kg grain M^−3^) compared with other treatments. The Mz–M system under PB + R increased IWP by 66% over the F-M under CT − R (farmers practice). The reported higher IWP in the CA-based maize-mustard system might be due to less water evaporation from the soil surface and higher moisture retention for longer periods under the residue cover. On the other hand, the frequent tillage in the CT plots may have resulted into more evaporative loss of soil moisture. Higher moisture retention in residue-based treatment helped proper germination/ emergence of mustard seedlings and stand establishment, better seedlings growth and, ultimately, higher yields of mustard. Crop residues led to better equilibrium between macro- and micro-(3-year mean) porosity, root development, biomass production, moisture content, yield and water productivity^27,33–35^.

### Soil organic carbon stock and nutrient productivity

Improvement in soil organic carbon (SOC) is a major goal of adopting CA practices. Conservation tillage practices significantly (p = 0.05) (Table 6) enhanced the soil organic carbon stock (Mg ha^−1^) compared with conventional tillage at two plow layers (0–15 and 15–30 cm) (Table 5). At 0–15 cm soil depth, PB + R (11.3 Mg ha^−1^) though, on par with ZT + R (10.6 Mg ha^−1^) increased SOC by 17.7% over CT − R (9.6 Mg ha^−1^) and 28.4% over the initial value (8.8 Mg ha^−1^). System intensification influenced SOC markedly at 0–15 cm soil depth and recorded the highest value in the GG-M system (12.1 Mg ha^−1^) which was followed by Mz–M (10.9 Mg ha^−1^) and CB-M (10.7 Mg ha^−1^). The lowest value (9.5 Mg ha^−1^) was recorded in the fallow-mustard system. The GG-M system increased SOC by 27.4% over fallow-mustard system and by 37.5% over the initial value (8.8 Mg ha^−1^) (3-year mean). SOC at 15–30 cm soil depth was lower when compared with that at the 0–15 cm depth, and was highest in PB + R (10.1 Mg ha^−1^). Conservation tillage practices (PB + R and ZT + R) increased SOC significantly over the CT − R by 29.5 and 19.2% (3-year mean), respectively. System intensification also increased SOC at 15–30 cm soil depth, with the highest value recorded in the GG-M system (10.0 Mg ha^−1^) followed by CB-M (9.4 Mg ha^−1^) and Mz–M (9.3 Mg ha^−1^). SOC stock increased in GG-M by 20.5% over the fallow-mustard (3-year mean). SOC stock improved in all the system at both the plow layers 0–15 cm and 15–30 cm over the initial value of 8.8 and 7.1 Mg ha^−1^, respectively. Soil structure and soil organic matter (SOM) are the two most dynamic soil properties, and are highly sensitive to agricultural management practices^36^. However, the detrimental effects of continuous tillage could be reduced by CA, which could accelerate soil aggregation and carbon sequestration^37^. Soil organic carbon as a soil health indicator influence the biological activity and soil suitability in terms of physical and chemical properties. Inclusion of legumes in the cropping system (green gram and cluster bean) in the present study increased SOC and PFPn might be due to increased N supply by their biological N fixation, and addition of low C:N ratio legume crop residue which decomposed easily and converted to soil carbon. Residue incorporation/retention helps in improving SOC through soil aggregate size and stability^26,38,39^. The minimum SOC was reported in the conventional tillage practices where repeated tillage and inversion of top soil might have resulted into faster soil organic matter degradation due to aggregate disruption, higher oxidation and mineralization, and nutrient loss^40^.

**Table 5:** Soil organic carbon stock and partial factor productivity of NPK influenced with CA-based Indian mustard systems (3-year mean).

Treatments^A^	Soil organic carbon (Mg ha^−1^)		Partial factor productivity (kg ha^−1^)		
	0–15 cm soil depth	15–30 cm soil depth	PFPn^B^	PFPp^C^	PFPk^D^
**Tillage practice**					
PB + R	11.3^a†^	10.1^a^	34.8^a^	42.4^a^	59.7^a^
ZT + R	10.6^a^	9.3^b^	31.9^b^	39.0^b^	55.1^b^
CT − R	9.6^b^	7.8^c^	31.0^b^	37.7^b^	53.3^b^
**Cropping system**					
F-M	9.5^d^	8.3^c^	34.9^c^	46.5^b^	69.8^b^
CB-M	10.7^b^	9.4^ab^	39.4^a^	39.4^c^	49.2^c^
GG-M	12.1^a^	10.0^a^	37.6^b^	37.8^d^	47.2^d^
Mz–M	10.9^b^	9.3^ab^	33.8^d^	48.2^a^	75.0^a^
PM-M	10.3^bc^	8.8^bc^	22.6^f^	29.0^e^	45.1^e^
S-M	9.9^ cd^	8.6^c^	27.1^e^	37.3^d^	49.8^c^

^†^Means followed by a similar lowercase letters within a column are not significantly different at 0.05 level of probability using DMRT. Initial SOC at 0–15 cm—8.8 and at 15–30 cm—7.1 Mg ha^−1^.

^A^Refer Table 7 for treatment description.

^B^Partial factor productivity of N.

^C^Partial factor productivity of P.

^D^Partial factor productivity of K.

Partial factor productivity (PFP) in terms of unit seed yield per unit of nitrogen, phosphorus and potassium applied, increased markedly (p = 0.05) (Table 6) under conservation tillage practices. PB + R, though, on par with ZT + R, recorded the highest partial factor productivity of N (PFPn) (34.8 kg ha^−1^), partial factor productivity of P (PFPp) (42.4 kg ha^−1^) and partial factor productivity of K (PFPk) (59.7 kg ha^−1^) which was 12.3, 12.5 and 12% higher over the CT − R, respectively (3-year mean) (Table 5). Intensification of fallow-mustard system with legumes (CB-M and GG-M) markedly increased the PFPn, whereas, PFPp and PFPk were higher under Mz–M system (3-year mean). PFPn recorded higher under CB-M (39.4 kg ha^−1^) followed by GG-M (37.6 kg ha^−1^) compared with fallow-mustard (34.9 kg ha^−1^). Whereas, intensification with maize, pearl millet and sesame in mustard-based systems recorded less PFPn compared with fallow-mustard system. The PFPp and PFPk were recorded highest under Mz–M system (48.2 and 75.0 kg ha^−1^) compared with fallow-mustard system (46.5 and 69.8 kg ha^−1^), respectively (3-year mean). Except Mz–M, other systems recorded less PFPp and PFPk compared with fallow-mustard system. The PFPn increased in CB-M and GG-M systems might be due to increased N supply due to biological N fixation, whereas, PFPp and PFPk recorded higher in Mz–M system might be due to incorporation of crop residues rich in P and K.

**Table 6:** ANOVA for ^a^soil organic carbon, ^b^partial factor productivity of N, ^c^partial factor productivity of P, and ^d^partial factor productivity of K.

Source	LSD (p = 0.05)					R^2^				
	SOC^a^ (0–15 cm)	SOC (15–30 cm)	PFPn^b^	PFPp^c^	PFPk^d^	SOC (0–15 cm)	SOC (15–30 cm)	PFPn	PFPp	PFPk
Tillage practices	0.80	0.60	1.93	2.18	2.91	0.82	0.89	0.98	0.98	0.99
Cropping systems	0.79	0.72	1.05	1.23	1.72					

## Conclusion

Indian mustard is mostly grown as rainfed crop under hungry and thirsty soils where excessive tillage operations liable to deplete soil fertility and productivity at faster rate, and make the system unsustainable. Conservation agriculture practices; reduced tillage, crop residue retention and crop diversification may sustain or increase crop productivity at reduced production and environmental costs, improve soil health and water use, and climate change resilience. Conservation tillage practices in mustard-based cropping systems achieved higher mustard yield as well as system-based input/output productivity and profitability. Intensification of conventional fallow-mustard system with maize-mustard system under CA based management (PB + R) proved to be a better alternative with respect to sustainability (+ 377%), production efficiency (+ 177%), economic efficiency (+ 94%) and water productivity (+ 66%). Conservation tillage and system intensification improved the SOC stock and NPK factor productivity compared with conventional fallow-mustard system which was fatigued with low SOC and PFPnpk due to higher tillage operations and no residue incorporation. Sustainable intensification of CT-based fallow-mustard systems through the CA-based maize-mustard system provides excellent opportunities to increase the system efficiency, farm income, soil health and simultaneously to reduce the government burden on import of edible oils. Combined, these results clearly demonstrate the potential of CA to simultaneously increase yield, diversify crop production and improve soil quality which should support a move towards sustainable intensification of crop production to improve future household income and food security.

## Methods

### Study site, climate and soil

**Figure 7:**
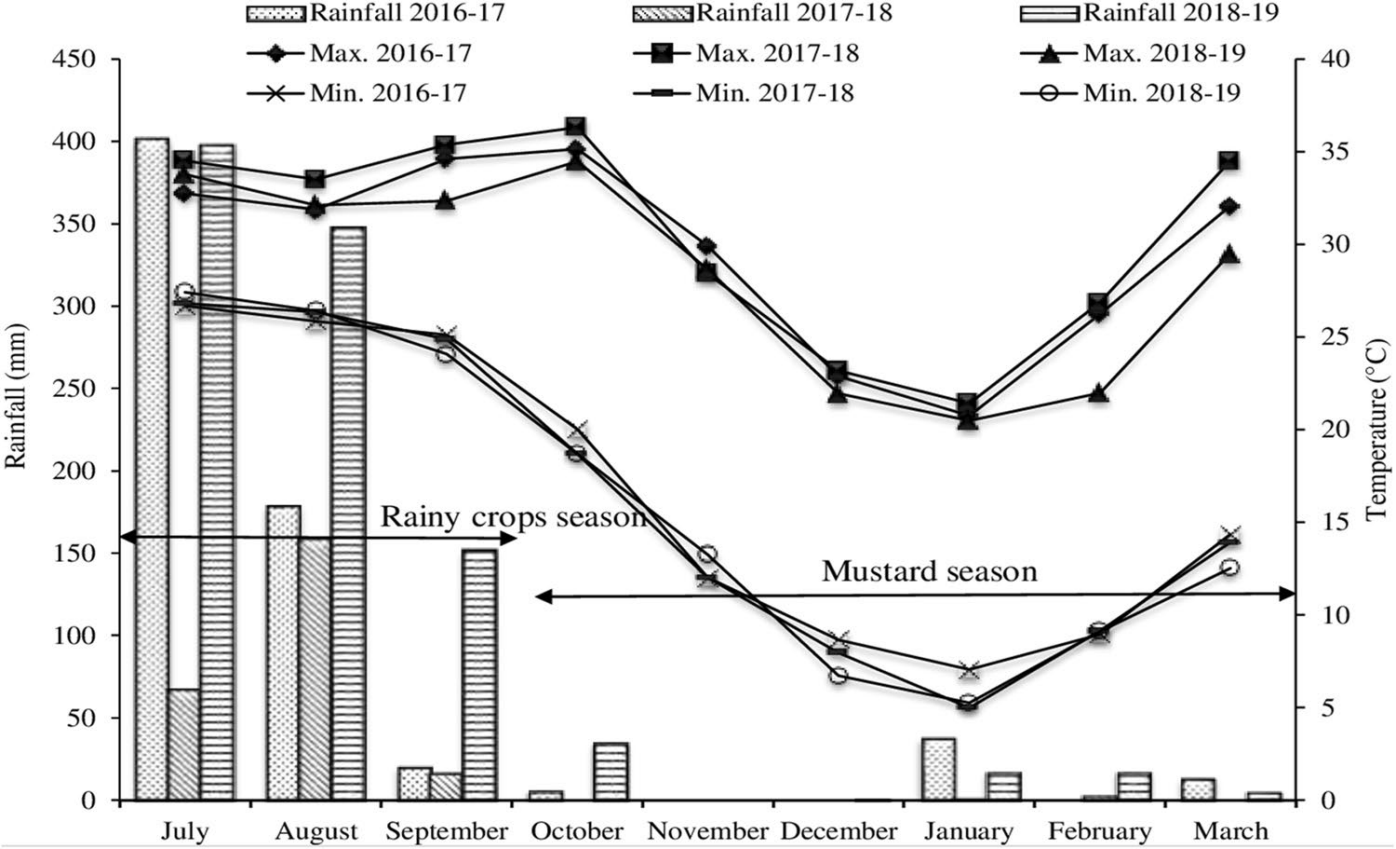
Monthly maximum and minimum temperature, and rainfall during the crop growing seasons (2016–2017, 2017–2018 and 2018–2019). Source: Agromet Observatory, ICAR-DRMR, Bharatpur, Rajasthan (India).

The experiment was conducted for 3-year (2016–2019) at the research farm, ICAR-Directorate of Rapeseed-Mustard Research, Bharatpur located at 77° 3′ E, 27° 15′ N and 178.37 m above mean sea level at same location and set of treatments. The climate is semi-arid, characterized with wide range of temperature between summer and winter. The meteorological observations were recorded daily and averaged to monthly during the crop growth period (July–September for rainy season crops and October–March for mustard crop) (Fig. 7). The maximum temperature during the crop growing season fluctuated between 20.5 in January and 36.3 °C in October, and minimum temperature between 7.0 °C January and 27.4 °C in July. The rainfall was mostly (75%) received during the south-west monsoon (July–September) and was recorded as 600, 242 and 898 mm during 2016–2017, 2017–2018 and 2018–2019, seasons, respectively. Winter rains were also received during the month of January which are very beneficial for the mustard crop growth and development at this stage. The soil pH and EC of the experimental site were 8.3 and 1.3 dS m^−1^, respectively. The soil samples were collected at the time of sowing and analyzed. The soils were poor in organic carbon (2.4 g kg^−1^) and available N (126.3 kg ha^−1^), while medium in 0.5 N NaHCO3 extractable P (17.2 kg ha^−1^) and 1.0 N NH4OAc exchangeable K (149.3 kg ha^−1^). The bulk density of the soil was 1.52 Mg m^−3^.

### Experimental design and treatments

In the present investigation, two factors (tillage practice and cropping systems with and without residue) were studied for three years in the split plot design. Three tillage practices were taken as main-plot factors to compare conservation tillage [Permanent beds with residue (PB + R) and zero tillage with residue (ZT + R)] with conventional tillage without residue (CT − R). Six cropping systems in rotation of rainy season crops with Indian mustard [fallow-mustard (F-M); cluster bean (*Cyamopsis tetragonoloba* L.)-mustard (CB-M); green gram (*Vigna radiata * L.)-mustard (GG-M); maize (*Zea mays* L.)-mustard (Mz–M); Pearl millet (*Pennisetum glaucum* (L.) R. Br.)-mustard (PM-M); and sesame (*Sesamum indicum* L.)-mustard (S-M)] were taken as sub-plot factors. The resultant 18 treatment combinations (3 × 6) were randomization and allocated as per design and replicated three times.

### Crop establishment

The experiment was initiated with deep plowing (30 cm) with chisel plough to break the hard pan and leveling of the soil surface. The rainy season crops were sown as per standard practices and according to treatments of interest (Table 7). The raised beds were prepared and planted the crop simultaneously in one operation with raised bed planter and seed-cum-fertilizer drill attachement. These beds were maintained for succeeding crops in cycle as permanent beds. In zero tillage plots, the crops were sown with zero till planter attached with seed-cum-fertilizer drill. The conventional tillage crops were sown after sequential tillage operations like harrowing (1-time), spring-tyne cultivator (5-time) and leveling (3-time) as the farmers’ practicing in the region.

**Table 7:** Treatment abbreviations and description of management protocols for different crops in Indian mustard-based cropping systems.

Crop rotation	Crop	Tillage			Crop establishment			Residue management		
		PB	ZT	CT	PB	ZT	CT	PB	ZT	CT
F-M	Mustard	One pass with permanent beds with planter	One pass zero till drill	Three passes of cultivator	Planted 2-rows on permanent beds (67.5 cm) at 30 × 15 cm spacing with multi-crop planter	Sowing with Zero till drill at 45 × 15 cm spacing	Sowing with seed drill at 45 × 15 cm spacing	30% retained	30% retained	Removed
CB-M	Cluster bean	One pass with permanent beds with planter	One pass zero till drill	Three passes of cultivator	Planted 2-rows on permanent beds (67.5 cm) at 30 × 10 cm spacing with multi-crop planter	Sowing with Zero till drill at 30 × 10 cm spacing	Sowing with seed drill at 30 × 10 cm spacing	10% retained	10% retained	Removed
GG-M	Green gram	One pass with permanent beds with planter	One pass of zero till drill	Three passes of cultivator	Planted 2-rows on permanent beds (67.5 cm) at 30 × 10 cm spacing with multi-crop planter	Sowing with Zero till drill at 30 × 10 cm spacing	Sowing with seed drill at 30 × 10 cm spacing	100% retained	100% retained	Removed
Mz–M	Maize	One pass of permanent beds with planter	One pass of zero till drill	Three passes of cultivator	Planted single row on permanent beds (67.5 cm) at 20 cm plant spacing with multi-crop planter	Sowing with Zero till drill at 60 × 20 cm spacing	Sowing with seed drill at 60 × 20 cm spacing	30% retained	30% retained	Removed
PM-M	Pearl millet	One pass of permanent beds with planter	One pass of zero till drill	Three passes of cultivator	Planted 2-rows on permanent beds (67.5 cm) at 30 × 10 cm spacing with multi-crop planter	Sowing with Zero till drill at 30 × 10 cm spacing	Sowing with seed drill at 30 × 10 cm spacing	30% retained	30% retained	Removed
S-M	Sesame	One pass of permanent beds with planter	One pass of zero till drill	Three passes of cultivator	Planted 2-rows on permanent beds (67.5 cm) at 30 × 10 cm spacing with multi-crop planter	Sowing with Zero till drill at 30 × 10 cm spacing	Sowing with seed drill at 30 × 10 cm spacing	20% retained	20% retained	Removed

The rainy season crops were sown in the first week of July after monsoon rains in all the three years. The cluster bean (cv. RGC 1003), green gram (cv. IPM 2–3), pearl millet (cv. RHB 173) and sesame (cv. HT 1) were planted at 30 cm row to row and 10 cm plant distance with a seed rate of 15, 12, 4 and 4 kg ha^−1^, respectively in the CT and ZT plots. While, these crops were planted in two rows at 18.75 cm spacing in PB plots. The maize crop (cv. QPM 1) was planted at a seed rate of 20 kg ha^−1^, at 67.5 cm row to row and 20 cm plant to plant distance in CT, ZT and PB plots. The dry season, Indian mustard was sown in the same plots as per the tillage treatments (CT, ZT and PB) after the harvest of rainy season crops in the cycle. Indian mustard var. RH 749 was used as test crop in all the years and planted in the first week of October at a seed rate of 4 kg ha^−1^ at 45 cm row to row and 15 cm plant to plant distance in the CT and ZT plots. Whereas, two rows of mustard were planted in PB plots at 18.75 cm row to row and 15 cm plant to plant distance. Each crop was accommodated in 15 × 6.4 m gross plot area and plant and soil observations were taken from 14 × 5.4 m net sown area of each treatment.

### Crop management

Both dry as well as rainy season crops were optimally nourished with their respective recommended doses of macro and micro nutrients. The recommended dose of N, P_2_O_5_ and K_2_O are 80, 40 and 40 kg ha^−1^ for Indian mustard; 120, 80 and 50 kg ha^−1^ for maize; 100, 80 and 50 kg ha^−1^ for pearl millet; 20, 40 and 40 kg ha^−1^ for green gram and cluster bean; and 30, 20 and 20 kg ha^−1^ for sesame, respectively. Per hectare 40 kg S, 5 kg Zn and 1 kg B to Indian mustard, and 5 kg Zn to maize were also applied. An additional dose of 20 kg N ha^−1^ was applied to PB and ZT treatments of Indian mustard to offset the effect of N immobilization. In the rainy season, cluster bean, green gram and sesame were fertilized with full amount of N, P_2_O_5_ and K_2_O at the time of sowing as basal application. Half dose of N and full dose of P_2_O_5_ and K_2_O as basal and remaining half N at the 30 days after sowing (DAS) was applied in pearl millet. Maize was fertilized with 1/3rd N and full dose of P_2_O_5_ and K_2_O and ZnSO_4_ at the time of seeding, while the remaining 2/3rd N was top dressed as equal splits at fifth leaf and tasseling stages. In the dry season, Indian mustard was fertilized with half dose of N and full dose of P_2_O_5,_ K_2_O, S, ZnSO_4_ and B at the time of sowing as basal application, and the remaining half N was top-dressed at the time of the first irrigation. Glyphosate at 1.0 kg a.i. ha^−1^ was sprayed 2-day prior to sowing in PB and ZT plots to control the weeds in both rainy and dry season. After sowing, atrazine at 1.0 kg a.i. ha^−1^ as pre emergence (PE) in pearl millet and maize; pendimethalin at 1.0 kg a.i. ha^−1^ as PE in Indian mustard, green gram and cluster bean; and alachlor at 1.5 kg a.i. ha^−1^ as PE in sesame were applied in all the plots (PB, ZT and CT). Additionally, one hand weeding was also done in CT plots for weed control at 30 DAS in all the crops.

The cluster bean, pearl millet, sesame and maize were harvested manually at maturity in the month of September. The green gram was harvested by hand picking of mature pods at three stages. All the rainy season crops were harvested by leaving 1/3rd crop portion on soil surface as anchored residue in the PB and ZT plots and remaining were removed for cattle feed and fodder. CT plots were harvested 5 cm above the soil surface without leaving any residue. At 75% siliquae maturity, the mustard was harvested in the month of March, leaving 1/3rd crop stubbles on soil surface as anchored residue in the PB and ZT plots and 5 cm above the soil surface without leaving any residue in the CT plots. Equal numbers of rows were harvested from a 14 × 5.4 m net sown area of each treatment in all the seasons and years.

### Recycling of crop residues

The mustard, cluster bean, green gram, maize, pearl millet and sesame were harvested from above the soil surface by leaving 30, 10, 100, 30, 30 and 20% crop portion as anchored stubbles in the field. Management protocols related to residue management are given in Table 8. In conventional tillage plots, 100% residue was removed.

**Table 8:** Total residue load under different tillage and cropping system over the years.

Cropping system	Residue retained (Mg ha^−1^)					
	Permanent beds			Zero tillage		
	Rainy crops	Mustard	System	Rainy crops	Mustard	System
F-M	0.0	2.3	2.3	0.0	2.4	2.4
CB-M	0.2	2.3	2.5	0.2	2.2	2.4
GG-M	1.6	2.2	3.8	1.3	2.4	3.7
Mz–M	1.9	2.3	4.2	1.5	2.2	3.7
PM-M	1.2	1.9	3.1	1.1	2.2	3.3
S-M	0.4	2.3	2.7	0.3	2.0	2.3

### Yield of crops and system grain yield

Equal numbers of rows of each crop were harvested manually from the net plot area (14 × 5.4 m), leaving anchored stubbles in the field as per treatment details (Table 8). The harvested produce was Sun-dried and threshed using mechanical thresher (maize grains separated with the sheller). The stubbles left over in the field of each crop were measured using 1.0 m^2^ quadrant at three places from the net plot and Sun-dried and added to the total stover yield crop-wise. The system productivity of different cropping systems were measured as mustard equivalent yield (MEY) by converting seed yield of rainy season crops to mustard using equation given below with an example of sesame.

$${\text{Mustard}}\;{\text{equivalent}}\;{\text{yield}}\left( {{\text{Mg}}\;{\text{ha}}^{{ - 1}} } \right) = \frac{{{\text{Sesame}}\;{\text{seed}}\;{\text{yield}}\left( {{\text{Mg}}\;{\text{ha}}^{{ - 1}} } \right) \times {\text{Sale}}\;{\text{price}}\;{\text{of}}\;{\text{sesame}}\left( {{\text{US}}\$ {\text{Mg}}^{{ - 1}} } \right)}}{{{\text{Sale}}\;{\text{price}}\;{\text{of}}\;{\text{mustard}}\left( {{\text{US}}\$ {\text{Mg}}^{{ - 1}} } \right)}}$$

### Net returns and relative economic efficiency

The economic profitability analysis was worked out for all the crops and cropping systems under the respective treatments. The total cost (TC) of cultivation includes all the input and related costs (field, labor, and electricity) that are involved in crop production from sowing to marketing. Gross returns (GR) were calculated by multiplying the crop yield with minimum support price that were offered by the Govt. of India, and the straw yield by current local market rates (Table 9). The net returns (NR) were calculated as the difference between the GR and the TC (NR = GR − TC). The system NRs were calculated by adding NRs of crops harvested within an individual calendar year. The prices of inputs and outputs are given in Table 9.

**Table 9:** Cost of key inputs and outputs used for economic analysis during different years.

Item/commodity	Price input and output ($ unit^−1^)		
	2016–2017	2017–2018	2018–2019
Mustard grain (kg^−1^)	0.53	0.57	0.60
Mustard straw (kg^−1^)	0.01	0.01	0.01
Cluster bean grain (kg^−1^)	0.50	0.53	0.55
Cluster bean straw (kg^−1^)	0.07	0.07	0.07
Green gram grain (kg^−1^)	0.74	0.79	0.99
Maize grain (kg^−1^)	0.19	0.20	0.24
Maize straw (kg^−1^)	0.03	0.03	0.03
Pearl millet grain (kg^−1^)	0.19	0.20	0.28
Pearl millet straw (kg^−1^)	0.03	0.03	0.03
Sesame grain (kg^−1^)	0.71	0.75	0.89
Sesame straw (kg^−1^)	0.01	0.01	0.01
Urea (50 kg^−1^)	4.19	3.80	3.80
Di-ammonium phosphate (DAP) (50 kg^−1^)	18.34	18.34	17.77
Single super phosphate (SSP) (50 kg^−1^)	4.55	4.55	5.12
Muriate of potash (MOP) (50 kg^−1^)	10.31	10.31	12.72
Zinc sulphate (ZnSO_4_) (5 kg^−1^)	4.69	4.98	6.97
Sulphur (S) (5 kg^−1^)	3.16	3.16	3.16
Wage rate (person^−1^ day^−1^)	4.38	4.56	4.93
US$ conversion rate	70.34		

#### Relative economic efficiency (REE)

The comparative advantages through tillage alterations and cropping systems were presented through REE and expressed in percentage^41^.

$${\text{REE}} = \frac{{\Delta {\text{NR}}}}{{\text{A}}} \times 100$$

where, ∆NR is the difference in the net returns from various tillage treatments over the control for main plots (CT) and the difference in net returns from various cropping systems over the fallow-mustard cropping system for subplots, A is the net returns from CT for main plots and the net returns from the fallow-mustard cropping system treatment for subplots.

#### Economic efficiency (EE)

The economic efficiency of various cropping systems under different tillage methods was calculated to determine per day return and calculated as:

$${\text{EE}} = \frac{{{\text{NR}}\left( {{\text{US}}\;\$ } \right)}}{{\text{D}}}$$

where, NR is the net returns in US $, and D is the duration of the crops in a system.

### Sustainability yield index (SYI)

The variation in mean seed yield (in terms of mustard equivalent yield) of each tillage practice and cropping system were compared with the maximum observed mustard equivalent yield over the years and expressed as sustainability yield index (SYI). It is expressed as;

$${\text{SYI}} = \frac{{{\text{Ya}} - \upsigma }}{{{\text{Ym}}}}$$

where, Ya is the mean seed yield (MEY), ${\upsigma }$ is the standard deviation of the yield, and Ym is the maximum seed yield (MEY) obtained under a set of management practices^42^.

### Production efficiency (PE)

Production efficiency (PE) represents the increase in seed yield on a daily basis. It is the ratio of total yield obtained during the crop period and duration of the crop^43^. The higher production efficiency indicates a better translocation of photosynthates from the source to the sink. It can be calculated by the following mathematical equation:

$${\text{PE}} = \frac{{{\text{Ya}}}}{{\Delta {\text{n}}}}$$

where, PE is the production efficiency (kg ha^−1^ day^−1^), Ya is the total grain yield (kg ha^−1^), and Δn is the total duration of the study (270 days).

### Water productivity

The amount of irrigation water applied to each plot was measured using a water meter. The total amount of water applied (input water) was computed by summing the irrigation (I) water and rainfall (R). The amount of irrigation water that was applied was quantified (mm ha^−1^) and calculated the irrigation water use productivity (IWP) as:

$${\text{IWP}} = \frac{{{\text{SY}}}}{{{\text{Wa}}}}$$

where, IWP is the irrigation water productivity in kg grain M^−3^ of water, SY is the seed yield (kg) and Wa is the water applied through irrigation (M^−3^).

### Partial factor productivity (PFP)

It is a simple production efficiency expression, calculated in units of crop yield per unit of nutrient applied. It answers to how productive a cropping system is, in comparison to its nutrient input.

$${\text{PFP}} = \frac{{\text{Y}}}{{\text{F}}}$$

where, PFP is the partial factor productivity (kg grain per kg nutrient applied), Y is the crop/system grain yield (kg ha^−1^) and F is the nutrient applied (kg ha^−1^).

### Statistical analysis

The data were subjected to analysis of variance for critical differences in split plot design using SSCNARS Portal online data analysis tool, IASRI (http://www.iasri.res.in/sscnars/2016). Calculated the simple effects, interaction effects and the least critical difference (p = 0.05) based on respective mean square errors. Then, Duncan Multiple Range Test was applied for grouping of significant or non-significant levels in main plot and subplot for ease of comparison of different levels within the factors and represented with small letters. The coefficient of determination (R-squared) was also calculated to show the per cent variability in the response data of a variable.

### Statement on guidelines

The experimental research and field studies on plants or plant parts used in the present study complies with the institutional guidelines.
